# Optimised plasma sample preparation and LC‐MS analysis to support large‐scale proteomic analysis of clinical trial specimens: Application to the Fenofibrate Intervention and Event Lowering in Diabetes (FIELD) trial

**DOI:** 10.1002/prca.202200106

**Published:** 2023-03-22

**Authors:** Matthew B. O'Rourke, Andrzej S. Januszewski, David R. Sullivan, Imre Lengyel, Alan J. Stewart, Swati Arya, Ronald C. Ma, Sanjeev Galande, Anandwardhan A. Hardikar, Mugdha V. Joglekar, Anthony C. Keech, Alicia J. Jenkins, Mark P. Molloy

**Affiliations:** ^1^ Bowel Cancer & Biomarker Lab School of Medical Sciences Faculty of Medicine and Health The University of Sydney Sydney Australia; ^2^ Centre for Inflammation Centenary Institute Sydney Australia; ^3^ School of Life Sciences Faculty of Science University of Technology Sydney Sydney Australia; ^4^ NHMRC Clinical Trials Centre Faculty of Medicine and Health The University of Sydney Sydney Australia; ^5^ Department of Chemical Pathology Royal Prince Alfred Hospital NSW Health Pathology Australia; ^6^ Wellcome‐Wolfson Institute for Experimental Medicine School of Medicine Dentistry and Biomedical Science Queen's University Belfast Belfast Belfast UK; ^7^ School of Medicine University of St Andrews St Andrews Fife UK; ^8^ Department of Medicine and Therapeutics The Chinese University of Hong Kong Hong Kong China; ^9^ Indian Institute of Science Education and Research Pune India; ^10^ Baker Heart and Diabetes Institute Melbourne Australia; ^11^ Present address: Diabetes and Islet Biology group School of Medicine Western Sydney University Campbelltown Australia

**Keywords:** Biomarker, diabetes, fenofibrate, mass spectrometry, plasma, proteomics

## Abstract

**Purpose:**

Robust, affordable plasma proteomic biomarker workflows are needed for large‐scale clinical studies. We evaluated aspects of sample preparation to allow liquid chromatography‐mass spectrometry (LC‐MS) analysis of more than 1500 samples from the Fenofibrate Intervention and Event Lowering in Diabetes (FIELD) trial of adults with type 2 diabetes.

**Methods:**

Using LC‐MS with data‐independent acquisition we evaluated four variables: plasma protein depletion, EDTA or citrated anti‐coagulant blood collection tubes, plasma lipid depletion strategies and plasma freeze–thaw cycles. Optimised methods were applied in a pilot study of FIELD participants.

**Results:**

LC‐MS of undepleted plasma conducted over a 45 min gradient yielded 172 proteins after excluding immunoglobulin isoforms. Cibachrome‐blue‐based depletion yielded additional proteins but with cost and time expenses, while immunodepleting albumin and IgG provided few additional identifications. Only minor variations were associated with blood collection tube type, delipidation methods and freeze–thaw cycles. From 65 batches involving over 1500 injections, the median intra‐batch quantitative differences in the top 100 proteins of the plasma external standard were less than 2%. Fenofibrate altered seven plasma proteins.

**Conclusions and clinical relevance:**

A robust plasma handling and LC‐MS proteomics workflow for abundant plasma proteins has been developed for large‐scale biomarker studies that balance proteomic depth with time and resource costs.

**Abbreviations**
FIELD: Fenofibrate Intervention and Event Lowering in Diabetes
QC: Quality control
STAGE tip: Stop and Go Extraction
Dep S: Immunodepletion of albumin and IgG
Dep G: Cibacron blue dye‐based depletion
HDL: high‐density lipoprotein
Apo‐AI: apolipoprotein AI
MEOCHL: methanol chloroform
MTBE: Methyl‐tert‐butyl‐ether

## 1 INTRODUCTION

Proteomics is increasingly used in clinical research to identify novel biomarkers for the prediction of health outcomes, including responses to interventions, suggest mechanisms of disease prediction and prevention, and facilitate the development of novel therapeutics [1, 2]. Such clinical studies are often extensive, and from heterogeneous subjects with potential impacts on proteomics analyses due to sample collection and handling [3, 4, 5]. Here, we set out to develop a robust, cost‐effective liquid chromatography‐mass spectrometry (LC‐MS) proteomic workflow to analyse large numbers of clinical plasma samples. Consideration was given to minimising the number of sample processing steps to control pre‐analytical variance while achieving detailed proteomic coverage of abundant plasma proteins. Given the large scale of many clinical studies, including the one which we will undertake, there is a need to establish a robust method for data quality for both sample preparation and subsequent mass spectrometric analysis, which would generally be acceptable for reporting undertaken clinical trials data.

Mass spectrometry‐based proteomic analysis is often performed in discrete, relatively small studies where cohorts of samples are prepared in a single instance and then measured in the mass spectrometer in a single batch. This ensures that variation introduced through sample handling techniques and instrument performance are kept at a minimum [6]. However, in the context of analysing thousands of plasma samples from clinical observational studies and trials, this is not feasible, requiring us to develop quality control (QC) methods and standardised protocols to ensure that each batch of samples could be obtained within a pre‐defined quantitative threshold. Some reports have provided blueprints for proteomic biomarker discovery workflows compatible with large‐scale studies. For example, Bruderer et al. [6] established a capillary LC‐MS/MS method with 300 μm I.D. columns to quantitate more than 1500 plasma samples, while Geyer et al. [7] quantified 319 plasma proteomes in quadruplicate. These papers provide excellent examples of specific LC‐MS workflows for plasma proteomics, but they remain demonstration studies for what can be achieved at scale in specialist labs and have not been widely implemented elsewhere. Moreover, important plasma sample characteristics and processing variables relevant to clinical studies were not explored in these reports.

This paper aims to share our experience in developing an LC‐MS proteomic workflow to enable robust quantitation of plasma proteins from extensive clinical studies. In designing the workflow, we assessed four critical variables related to plasma collection and processing for proteomics LC‐MS analyses: (1) depletion of abundant plasma proteins compared with neat plasma analysis, (2) approaches for plasma delipidation, (3) EDTA plasma compared with citrate plasma and (4) impact of sample freeze–thaw cycles. We report a proof‐of‐concept pilot study that includes using the optimised workflow with plasma samples from 15 Fenofibrate Intervention and Event‐Lowering in Diabetes (FIELD) [8] trial participants pre‐ and post‐16 weeks of fenofibrate treatment, as well as our subsequent quantitative results using an external plasma standard measured over an 18‐month period by DIA LC‐MS across 65 batches of 24 plasma samples per batch.

## 2 MATERIALS AND METHODS

### 2.1 Materials

A citrated plasma sample was used as an external standard for batch QC assessment (Sigma–Aldrich, North Rocks, Sydney, Australia). Methyl‐*tert*‐butyl‐ether (MTBE), methanol, acetonitrile (ACN), acetone, ethyl acetate, ammonium hydroxide, formic acid (FA), Tris‐HCl pH8.0, chloroacetamide (CLA), tris(2‐carboxyethyl)‐phosphine (TCEP), monopotassium phosphate, triethylammonium bicarbonate (TEAB), chloroacetamide, sodium deoxycholate (SDC) and trifluoroacetic acid (TFA) were all of ultra‐pure or LC‐MS grade and purchased from Sigma–Aldrich. ProteoPrep Immunoaffinity Albumin and IgG depletion Kits were from Sigma–Aldrich). Aurum Affi‐Gel Blue Mini Columns were from BioRad (Hercules, CA, USA). Sequencing grade trypsin was from Promega (Madison, WI, USA), and iRT peptides were from Biognosys (Schlieren, Switzerland). SDB‐RPS disks were from Affinisep (Petit‐Couronne, France).

### 2.2 Subjects

The study was conducted according to the guidelines of the Declaration of Helsinki and approved by the Human Research Ethics Committee of The University of Sydney (08‐2007/10216). All participants provided written informed consent.

For the experiments comparing abundant protein depletion, lipid depletion methods, blood collection tubes and assessing effects of sample freeze–thaw, we obtained plasma from five healthy volunteers aged between 27 and 59 years, three men, two women, mean age 40 years (±13 years standard deviation).

In a pilot study, citrate plasma samples from 15 FIELD trial participants pre‐ and post‐16‐week run‐in during the last 6‐weeks of which once daily fenofibrate tablets (200 mg comicronised) were taken. Subsequently, we report the inter‐assay CVs of the external plasma standard used for batch QC in 72 runs of batches of 24 FIELD samples.

### 2.3 Albumin and immunoglobulin depletion

The Australian Proteome Analysis Facility (Macquarie University, Sydney, Australia) performed the depletion of abundant proteins as per manufacturer's instructions. For immunoaffinity depletion of albumin and IgG, ProteoPrep immunoaffinity depletion columns were equilibrated in loading buffer (20 mM sodium phosphate, 150 mM NaCl, pH 7.4) and 50 μL of neat plasma added. Samples were incubated in the columns for 15 min at room temperature, followed by washing twice, with 400 μL loading buffer. The washes were combined and stored at −80°C as these contained the unbound plasma proteins. The bound fraction was discarded. For Cibacron Blue based depletion, an Aurum Affi‐gel Cibacron Blue F3GA agarose gel microcolumn was used following the manufacturer's instructions. Briefly, columns were drained, and washed with loading buffer (20 mM sodium phosphate, pH 7.4). 125 μL of neat plasma diluted in 375 μL of loading buffer was then added to the column and spun using a benchtop centrifuge at 10,000 × *g* for 20 s. This procedure was repeated with another 400 μL of loading buffer, and the eluates were combined and stored at −80°C.

### 2.4 Processing of un‐delipidated plasma

Plasma tryptic digests were prepared by transferring 10 μL of plasma to a 1.5 mL Eppendorf tube and adding 800 μL of 1% w/v SDC in 100 mM TEAB and vortexed. 100 μL of diluted plasma was then removed to a fresh tube, and 1 μL of 1 M TCEP was added together with 4 μL of 1 M CLA. Samples were boiled at 95°C for 10 min. Next, microgram of trypsin was added, and samples were digested overnight at 37°C.

Following digestion, 1 μL of FA was added to precipitate the SDC, and the tubes were centrifuged at 18,000 × *g* in an Eppendorf benchtop microfuge for 5 min. The resulting supernatant was then desalted using the Stop and Go Extraction (STAGE Tip) desalting method below.

**Statement of Clinical Relevance**

Plasma biomarkers hold great promise to improve diagnosis and influence aspects of clinical practice, but their discovery is hampered by analytical challenges in study design and specimen analysis. Clinical studies require large‐scale analysis of hundreds of specimens, so fit‐for‐purpose proteomic workflows that balance throughput and cost while ensuring quantitative rigor are required. Our aim was to evaluate human plasma sample suitability and preparation procedures for liquid chromatography‐mass spectrometry (LC‐MS) which addresses these challenges. We determined that using neat, citrated plasma with online delipidation over 45 min of LC‐MS sample acquisition time was a cost‐effective way to quantify 172 abundant plasma proteins with high quantitative reproducibility. We report the intra‐batch variability across 65 batches to be less than 2%. This demonstrates a viable approach to support large‐scale clinical biomarker studies using human plasma.

### 2.5 Offline delipidation

Three different methods of offline delipidation were tested.

#### 2.5.1 Chloroform–methanol extraction

Plasma (10 μL) was aliquoted into a clean 1.5 mL Eppendorf tube, and then 50 μL of cold Milli‐Q H_2_O was added. Next, 75 μL of 1 M monopotassium phosphate, 125 μL of methanol, 375 μL of chloroform and 150 μL of cold water were added to the plasma, followed by 2 min of high‐speed vortexing. Samples were then centrifuged at 18,000 RCF to remove the aqueous and organic phases (top and bottom liquid fractions). The tube was then dried in a vacuum desiccator for 5 min to remove any residual solvent or water. The protein pellet was then resuspended in 200 μL of 1% SDC 100 mM TEAB, and a 10 μL aliquot was removed for BCA protein assay. The sample was processed with trypsin and peptides desalted as described above.

#### 2.5.2 Acetone precipitation

Acetone precipitation of proteins was performed by taking 10 μL of plasma and adding 1 mL of ice‐cold acetone. Samples were then left at −20°C for 1 h before centrifuged at 18,000 × *g* for 5 min to pellet the precipitated protein. 200 μL of 1% SDC 100 mM TEAB was added to solubilise the protein pellet before 10 μL was removed for BCA protein assay. The sample was processed with trypsin and peptides desalted as described above.

#### 2.5.3 Methyl‐*tert*‐butyl‐ether extraction

Neat plasma (84 μL) was transferred into a 1.5 mL tube, and then 100 μL of methanol and 340 μL of MTBE were added. The sample was vortexed at high speed for 2 min, then centrifuged (18,000 × *g*, 5 min) to separate phases before discarding both phases from the tube and drying the protein pellet. The dried pellet was resolubilised in 200 μL of 1% SDC 100 mM TEAB and 2 μL of TCEP, 8 μL of CLA added, and the sample boiled (10 min at 95°C). Trypsin was added in a 1:100 ratio and digested overnight before STAGE Tip desalting.

### 2.6 STAGE Tip processing

As described previously, a combined STAGE Tip digestion/desalting/delipidation workflow was conducted [9]. Following tryptic digestion, 250 μL of 99% ethyl acetate, 1% TFA was added to each sample and vortexed for 10 s. Samples were then processed in a multiplex batch utilising a 3D printed apparatus described in Harney et al. [10].

### 2.7 Freeze–thaw cycles

EDTA plasma samples (1.2 mL in triplicates) from three volunteers were aliquoted (200 μL) into six separate tubes and frozen at −80°C. Five tubes per subject were then removed from the freezer and allowed to thaw on a roller mixer at room temperature for 30 min, then refrozen at −80°C. Samples remained at −80°C for 1 day before the next cycle. This procedure was continued stepwise until the final tubes were frozen and thawed six times. Each sample was prepared using the STAGE Tip, method and LC‐MS data collected in DDA mode as described below.

### 2.8 Blood collection tubes

Matched K2 EDTA and sodium citrate plasma from five volunteers were processed as per the STAGE Tip methodology described above. LC‐MS data was collected in data‐independent acquisition (DIA) mode as described below.

### 2.9 Liquid chromatography‐mass spectrometry (LC‐MS) analysis

LC‐MS was conducted with 2 μg plasma tryptic digest, separated using a self‐packed 150 μm I.D. × 15 cm column containing 1.9 μm C18 beads (ReproSil‐Pur C18‐AQ) on a 45 min gradient to a maximum of 30% B (80% ACN 0.1% FA), (63 min method runtime) using an Ultimate 3000 RSLCnano system (Thermo Scientific) operating at capillary flow rate of 1.6 μL/min. Data was acquired using a Q Exactive HF mass spectrometer (Thermo Scientific).

For experiments investigating impacts of abundant protein depletion, delipidation and freeze–thaw cycles, a Top15 data‐dependent acquisition (DDA) mode was used on Q Exactive HF mass spectrometer, employing using the same chromatography, columns and run‐length as DIA experiments. The following instrument settings were applied for DDA experiments: MS1‐AGC 3e6, resolution 60 K, scan range 300–1650 *m*/*z*. MS2‐AGC 1e5, resolution 15 K, loop count 15.

For samples prepared using the STAGE Tip workflow, DIA mode using variable m/z windows over the 300–1600 *m/z* range was used as previously described [9].

### 2.10 Proteomic data analysis

For LC‐MS data acquired using DDA, raw files were searched with MaxQuant V1.6.5 against the human proteome FASTA database (UP000005640, 20,350 entries) using the following settings: 1% FDR, reverse decoy mode, min peptides 1, FTMS mass tolerance 20 ppm, missed cleavages set to max 2. Modifications: oxidation of methionine, variable and carbamidomethyl (C) fixed, LFQ enabled with default settings.

LC‐MS data acquired using DIA were analysed using Spectronaut Pulsar X v12.0.2 (Biognosys, Switzerland) following our methods using an enhanced spectral library derived from publicly accessible human plasma datasets as we previously described [9]. Raw files were searched in Spectronaut against the spectral library [9] with the following settings: XIC extraction dynamic, precursor *Q*‐value cutoff value 0.01, protein *Q*‐value cutoff value 0.01. Peptides were quantified by a minimum of three transitions, protein quantification by top *n* = 5 peptides. Data were then exported as a ‘*Q*‐value Sparse’ pivot report matrix with normalised protein areas. Missing values were represented with ‘filtered’. Perseus V1.6.1.2 [11] was used to log2 transform data, and missing values were imputed using the following strategy: random values within 30% of the median of the normal distribution were selected, followed by a reduction by three standard deviations. To compare the two groups, Student *t*‐tests were used with *p* < 0.05, considered significant. To compare multiple groups, single factor analysis of variance (ANOVA) was used with *p* < 0.05 considered significant. For intra‐batch analyses the top 100 most abundant proteins from the external plasma standard were assessed for their pre‐ and post‐batch area differences. The median area differences for each batch were recorded.

## 3 RESULTS

### 3.1 Abundant protein depletion

**FIGURE 1:**
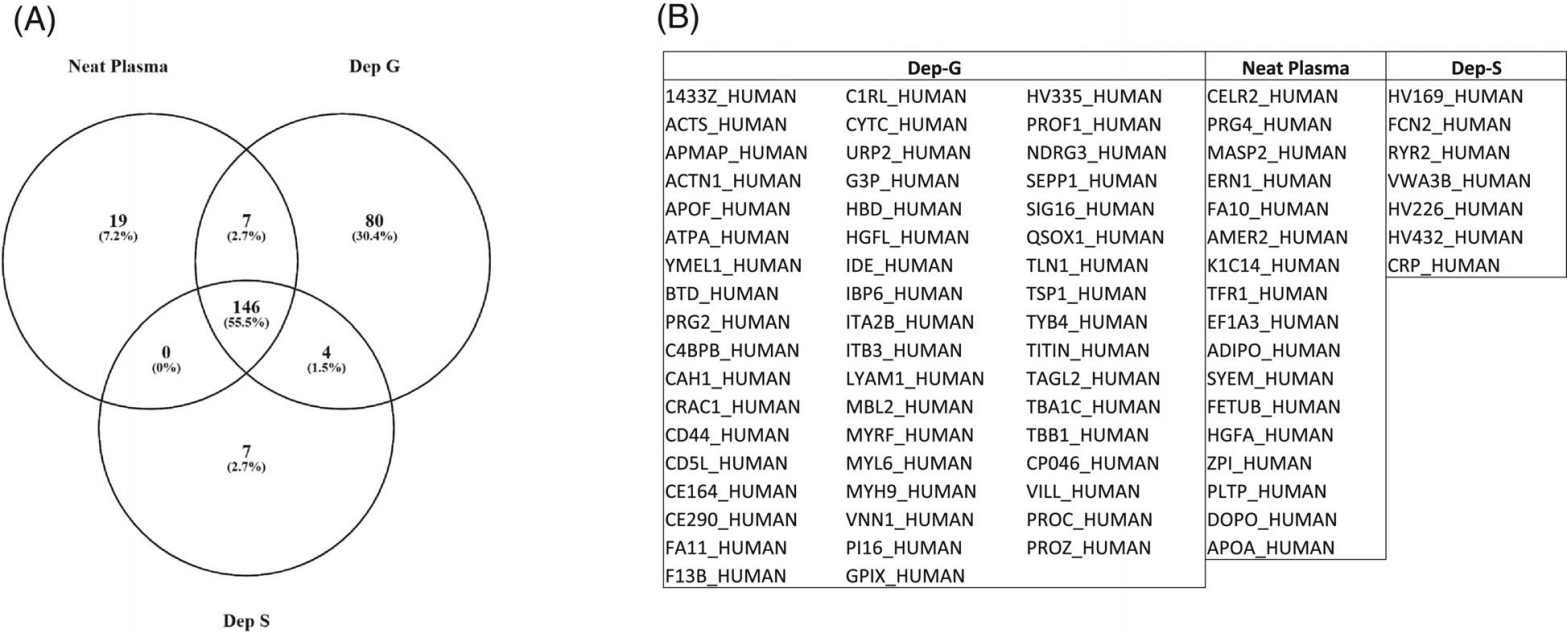
(A) Venn diagram showing a comparison of the number of proteins identified using different depletion methods. Dep G represents samples depleted with Cibacron blue dye, Dep S represents samples depleted serum albumin and IgG. (B) List of unique proteins detected using each method (immunoglobulin chain proteins are not shown).

It is well‐established that the high concentration of some proteins in plasma, such as immunoglobulins and albumin, limits the depth of proteome analysis that can be achieved in a single run LC‐MS experiment [12, 13]. Affinity‐based depletion methods have been used to remove these highly abundant proteins to address this high dynamic range issue [14]. However, for large‐scale studies as proposed here, added costs and time delays arising from using depletion would need to be justified. Therefore, we assessed the depth of proteome analysis that could be achieved with neat plasma compared with two affinity depletion approaches; specifically immunodepletion of albumin and IgG (Dep S) and non‐specific Cibacron blue dye‐based depletion (Dep G). Using a 45 min LC gradient and single‐shot top 15 DDA method, we detected a core group of 146 proteins in all three conditions. The use of Dep G revealed an additional 80 unique proteins, Dep S had seven unique proteins, while neat plasma showed 19 unique proteins (Figure 1 and Supplementary Data 1). When this was further investigated, Dep G resulted in a high number of additional immunoglobulin protein identifications, with 27 being attributable to heavy and light chain variable regions. A smaller subset of proteins was found when immunoglobulins were discounted from depleted and non‐depleted data (see Figure 1B). Immunodepletion with Dep S offered no tangible advantage to increasing proteome access compared with use of neat plasma. Dep G provided access to 40 additional proteins over neat plasma, although this comes with additional costs and sample handling steps. We detected apolipoprotein AI (Apo‐AI) in neat plasma, and it appeared to be depleted in the two other methods. This is a relevant finding as Apo‐AI, a key component of high‐density lipoprotein (HDL), is a biomarker of the lipid drug fenofibrate activity. We, therefore, considered that analysis using neat plasma is the most facile approach that affords time and cost advantages and would minimise quantitative variability due to sample handling. This came at the expense of 40 fewer proteins that could be detected using Dep G.

### 3.2 Plasma delipidation methods

Given that plasma contains numerous lipid species that may accumulate on stationary phase [15] and having selected neat plasma as the starting matrix, we tested various delipidation procedures with the objective to maximise LC column and MS system stability needed to analyse hundreds of clinical samples. We tested four delipidation methods: acetone, MTBE, methanol chloroform (MEOHCL) and ethyl acetate integrated into the STAGE Tip purification protocol [16].

**FIGURE 2:**
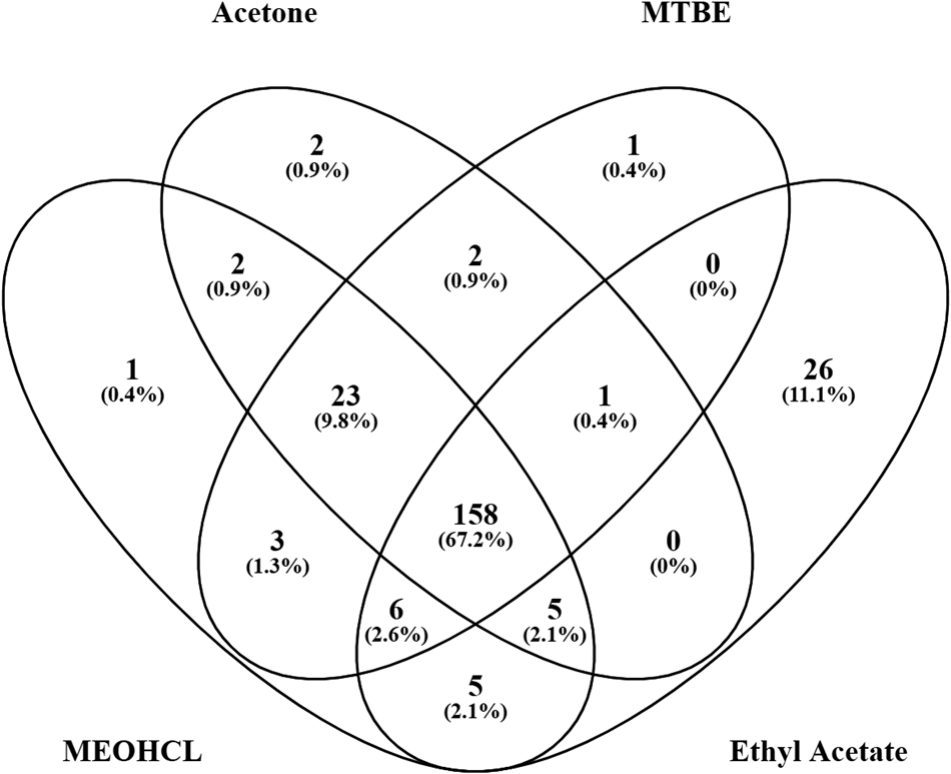
Venn diagram comparing number of proteins recovered following various delipidation methods. Methyl‐*tert*‐butyl‐ether (MTBE), methanol chloroform (MEOHCL), acetone and ethyl acetate Stop and Go Extraction (STAGE Tip) protocol.

We used DDA to access the recovery of proteins from triplicate preparations of each method (Figure 2). Unsurprisingly, we observed a high overlap amongst the methods, although interestingly, ethyl acetate integrated with STAGE Tip desalting led to the recovery of 26 unique proteins.

Similarly, the three other extraction methods recovered 23 proteins absent from the ethyl acetate STAGE Tip purification (Supplementary Data 2). This demonstrates that the selection of delipidation strategies has a minor effect on protein identifications. Of the methods tested herein, we chose to adopt the ethyl acetate integrated workflow for its simplicity in manual handling that is compatible with 96‐well plate‐based processing of samples.

### 3.3 Comparison of blood collection tubes

Most plasma proteomic studies utilise EDTA plasma, and there is little information regarding comparisons with sodium citrate plasma. This is relevant to the FIELD study as only citrate plasma was available for the proteomic analyses. There were no significant differences in the protein quantitation between plasma sample types, except five proteins (Table 1, Supplementary Data 3).

**TABLE 1:** Differentially abundant proteins in EDTA and sodium citrate plasma samples.

Protein accession number	Protein names	Protein descriptions	AVG Log2 ratio EDTA versus citrate	*Q*‐value
P02675	FIBB_HUMAN	Fibrinogen beta chain	−0.59	1.05E‐42
P02671	FIBA_HUMAN	Fibrinogen alpha chain	−0.59	3.92E‐40
P02751	FINC_HUMAN	Fibronectin	−1.69	3.02E‐39
P68871	HBB_HUMAN	Haemoglobin subunit beta	2.21	5.52E‐37
P69905	HBA_HUMAN	Haemoglobin subunit alpha	2.18	3.50E‐21

We observed higher abundance of haemoglobins in EDTA plasma, while citrate plasma showed higher abundances of fibrinogen and fibronectin [17].

### 3.4 Effects of multiple freeze–thaw cycles on stored plasma

**FIGURE 3:**
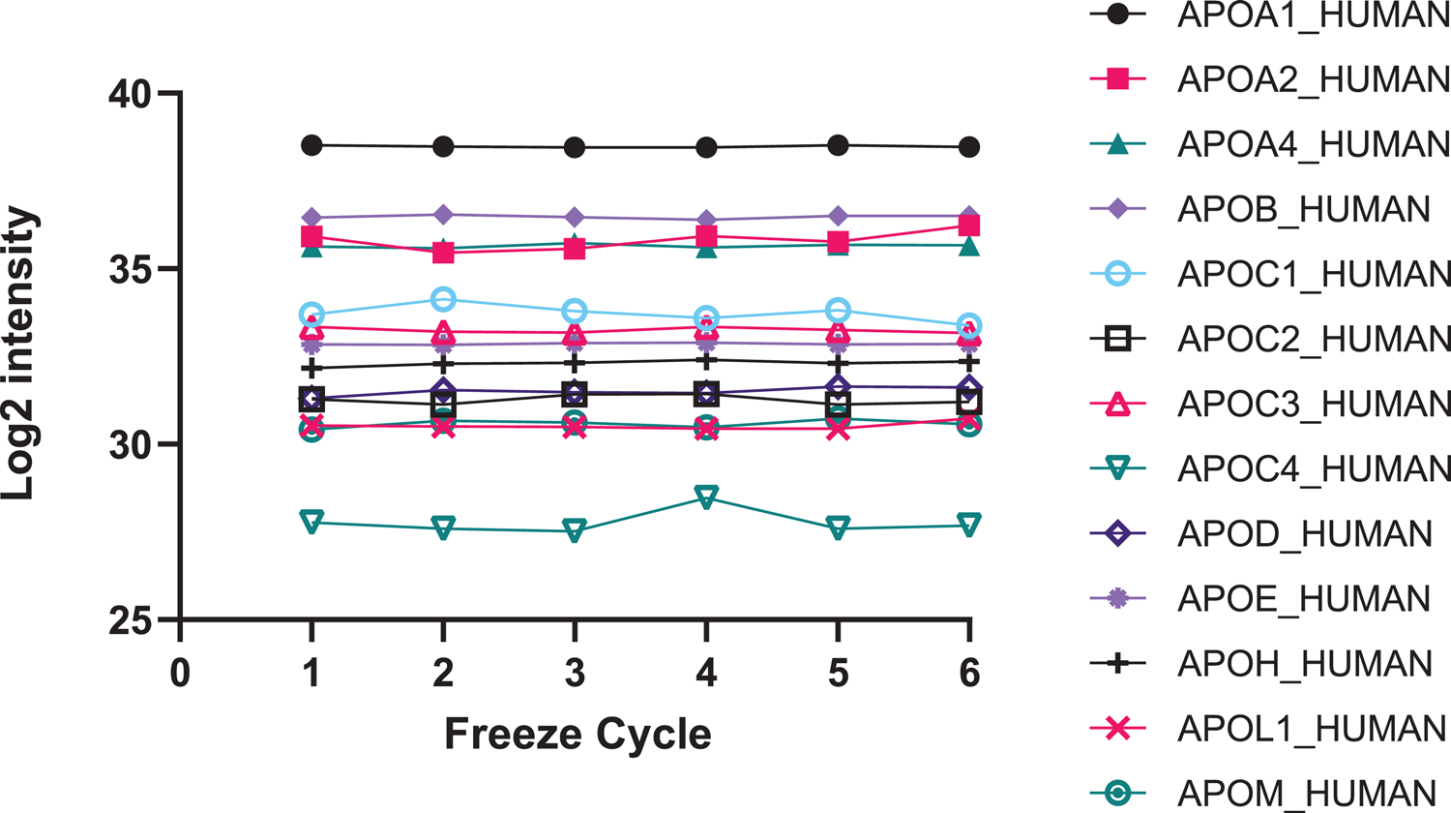
Relative abundances of apolipoproteins are stable across six freeze–thaw events.

We conducted sample freeze–thaw events to assess the effects of six freeze–thaw cycles on the quantitation of proteins. Overall, there was no significant variation in protein abundance between the cycles (ANOVA on 270 proteins was non‐significant) (see Supplementary Data 4). To further illustrate this, Figure 3 shows the relative abundance of apolipoproteins, displaying minimal variance across six freeze–thaw events.

### 3.5 A pilot study of FIELD samples

We applied the above determined optimal conditions, using neat, sodium citrate plasma with ethyl acetate STAGE Tip delipidation and DIA LC‐MS strategy to a cohort of 15 FIELD trial participant's plasma samples obtained before and after active run‐in (6 weeks fenofibrate treatment). A total of 222 proteins were detected across each of the 30 plasma samples (Supplementary Table S5). A differential analysis comparing pre‐ and post‐treatment groups identified seven significantly differentially regulated proteins (*p* < 0.05) (see Table 2). The major HDL protein constituents, Apo‐AI and Apo‐AII, showed a non‐significant increase post‐6‐weeks of fenofibrate in this small cohort. There was a significant decrease in the abundance of kallistatin (log_2_FC −0.13, *p* = 0.04), a serine proteinase inhibitor that some studies suggest is a biomarker for microvascular complications in diabetes patients [18].

**TABLE 2:** Showing significantly regulated proteins in 15 patients treated with fenofibrate (complete protein information can be seen in Supplementary Data 5)

Protein accession number	Protein name	Log_2_ FC post–pre	*p*‐value
P02787	Serotransferrin	0.18	0.007
B9A064	Immunoglobulin lambda‐like polypeptide 5	−0.17	0.017
A0A0C4DH24	Immunoglobulin kappa variable 6–21	−1.97	0.022
P04003	C4b‐binding protein alpha chain	0.20	0.024
P04350	Tubulin beta chain	2.66	0.033
P20742	Pregnancy zone protein	−0.21	0.037
P29622	Kallistatin	−0.13	0.040

### 3.6 Quantitative reproducibility in large scale analyses

**FIGURE 4:**
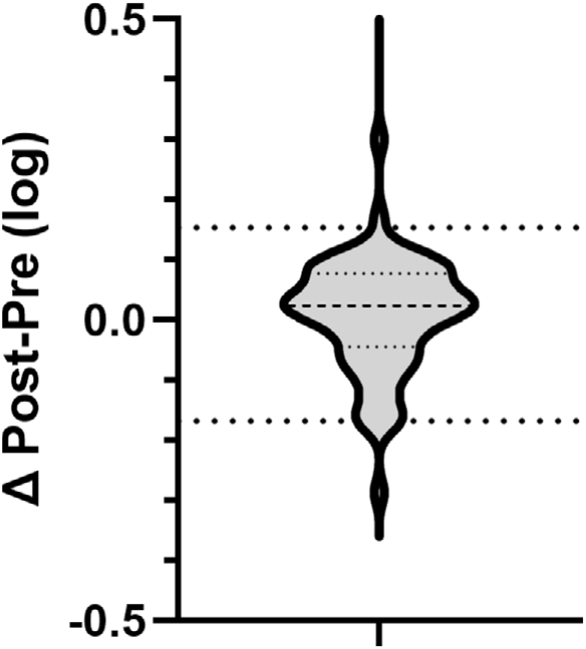
Intra‐batch protein abundance differences of external standard (post‐batch versus pre‐batch) for top 100 proteins in 65 batches. Major dash – median, minor dashes 25% and 75%. Major dots – 5% and 95%.

The above methods were applied to a cohort of FIELD trial participants (1560 samples data to be reported elsewhere). Here, we report a summary of the intra‐batch quantitative reproducibility associated with LC‐MS analysis of the external QC plasma standard. The external plasma standard was acquired pre‐ and post‐65 batches of 24 samples and the difference in areas of the top 100 most abundant proteins used to assess batch acceptability. The median intra‐batch difference was 1.6%, with the medians for 95% of the data within the range −9%–11%. (Figure 4 and Supplementary Data 6).

## 4 DISCUSSION

Our objective was to evaluate plasma sample handling workflows to optimise a protocol for the large‐scale analysis of human plasma for clinical studies which could be used for subsequent analyses of FIELD trial samples. Key factors evaluated included the reproducible quantitative measurement of protein data with appropriate QC measures, high throughput capability and a simplified sample preparation protocol to minimise variability caused by operator processing. To achieve this, we explored the capabilities of several sample preparation techniques. We applied QC measures to ensure stable instrument performance and low sample processing variability that could be achieved across a cohort of over 1560 human samples.

### 4.1 Removal of highly abundant plasma proteins

It has been previously established that there is an advantage to using top *N* abundant protein depletion for increasing the detectible proteome in plasma when conducting conventional nano‐LC‐MS experiments [19]. Our investigations using 45 min LC gradient with capillary flow and with DDA mode acquisition demonstrated a moderate increase in the number of detected proteins can be achieved following non‐selective partitioning using Cibacron blue dye (Figure 1). At the same time, specific depletion of albumin and IgG afforded no significant benefit in our LC‐MS/MS workflow. Several factors may contribute to this finding including incomplete depletion given the plasma concentration of albumin and IgG is extremely high and there is considerable inter‐patient heterogeneity in disease states, non‐specific binding causing inter‐sample quantitative variability, while other detractive aspects include additional sample handling steps and high reagent costs. Taken together, we failed to find sufficient evidence that plasma depletion combined with single‐shot LC‐MS acquisition offered benefit compared to the use of neat plasma in our workflow designed to robustly quantitate the most highly abundant plasma proteins in thousands of samples.

The consumable and data acquisition cost of running a single plasma sample in our laboratory is currently ∼$20 AUD. This includes a budget for instrument time, reagents and related consumables. When applied to the FIELD cohort of 1690 samples (1560 plasma samples + 130 QC samples), the projected cost of the study was exceeding $30,000 AUD. The Dep G depletion at $15 AUD per sample would add an additional $23,400 AUD in experimental costs for this study without a considerable gain in proteomic depth. Non‐specific depletion methods such as Dep G may further compromise an investigation by depleting potentially important biomarkers, such as Apo‐AI in our case, a known biomarker for fenofibrate response and potential modulate of diabetes complications. In this study, we demonstrated that undepleted, neat plasma could be effectively analysed by LC‐MS on a large‐scale while maintaining high‐quality instrument performance, and this strategy avoids complications associated with partial or incomplete depletions. The limitation to this approach is proteome depth, which can only be addressed using more extensive sample fractionation methods and come with added time and cost impacts.

### 4.2 Evaluation of delipidation methods

Human plasma contains as much as 471 mg/dL weight of lipids primarily sterol lipids, glycerophospholipids, glycerolipids, sphingolipids amongst other classes and species [20]. Moreover, large, hydrophobic lipids may be retained on reversed‐phase LC columns and contribute to poor reproducibility [15] and column blocking [10] so we rationalised we should deplete these to meet our objective for a robust and stable LC‐MS system to quantitate thousands of clinical specimens. Three different offline lipid removal methods were trialled, including one‐step protein precipitations using acetone, and, more complex workflows, which separate lipids and proteins by inducing phases in the aqueous and organic layers. A drawback of the two‐phase methods is the need to carefully pipette around the protein pellet, which sits at the interface of both phases, to remove the aqueous and organic phases. The use of offline methods of delipidation also creates a need to perform offline desalting. The manual handling required to perform these two separate steps of the workflow adds to the potential for introducing quantitative bias. We also trialled an online method of delipidation and desalting in a single multiplexed workflow. The Mann group published the STAGE Tip method in 2003 [16] is now a well‐established method for desalting peptide samples. Harney et al. [10] further adapted it for high‐throughput analysis by using a custom‐designed 3D printed tip holder in microplate format, enabling parallel analysis of 96 samples.

We found that the online delipidation method was the most convenient for handling multiple samples. With the aid of a combination style repeating pipette (e.g., Eppendorf Combipette M4), the ability to prime, wash and elute peptides was rapid and reproducible. One downside of the online method is that the solvents used for washing and elution are highly volatile and hazardous, meaning that venting had to be employed to remove vapours following centrifugation. The performance of our workflow without a delipidation step was not accessed.

### 4.3 Blood anti‐coagulants

Most plasma proteomic studies deploying LC‐MS utilise samples collected into EDTA anti‐coagulant tubes [21, 22]. There is a paucity of proteomic studies examining plasma proteins collected from other types of anti‐coagulant tubes. This is relevant to the FIELD study where citrated plasma samples are available for specimens collected over 12 years ago [8]. We performed a pairwise comparison of matched citrate and EDTA preserved plasma from a group of donors and observed minimal quantitative differences except for haemoglobin, fibrinogen and fibronectin levels. The suspected antagonistic nature of fenofibrate on cardiovascular complications has been linked to fibrosis related cardiovascular remodelling [23]; therefore, the elevated detection of these clotting related proteins when using citrate was deemed useful. Contrastingly the use of EDTA has been previously reported to increase the osmotic fragility of erythrocytes leading to increased haemolysis [24]. When EDTA was used, we found that elevated levels of haemoglobin were encountered, consistent with minor red blood cell haemolysis, which was absent in the citrated samples. This data confirmed the utility of sodium citrate plasma for LC‐MS proteomic analyses.

### 4.4 Effects of multiple freeze–thaw cycles on stored plasma

One of the considerations of this study was the effect of multiple freeze–thaw cycles. Parallel mRNA analyses were also planned for the specific cohort of chosen samples, so it was important to determine which analysis would need to take priority. RNA is typically less stable and prone to degradation when frozen and thawed multiple times; therefore, the effects of various freeze–thaw cycles were evaluated to see the impact on the detectible proteome. A subset of (non‐FIELD study) donor plasma was collected, and aliquoted, before being frozen and thawed six times. After observing no significant global variation, we chose a subset of apolipoproteins and complement proteins for this analysis. Our results showed that there was little effect on the abundance or detection of these markers of fenofibrate action. We attribute this to the denaturing conditions used for protein digestion, meaning that if proteins were degraded by freeze–thaw, this might not have been detectable in the subsequent LC‐MS peptide analysis. We note that it has previously been reported that the concentration of plasma proteins remains stable when specimens are stored at −80°C [25].

### 4.5 FIELD sample pilot study

To evaluate the effectiveness of the optimised workflow, a pilot study was conducted using stored FIELD study citrate plasma samples from patients treated with fenofibrate for 6 weeks, with fasted venous blood being taken pre‐ and post‐treatment. Minor increases in the major HDL lipoproteins Apo‐AI and Apo‐AII were observed after 6‐weeks of fenofibrate, although this trend did not reach statistical significance in this small cohort. Despite the small cohort size, several proteome changes were observed, including a change in the amount of circulating kallistatin reported previously as being associated with cardiovascular complications in people with diabetes [18]. While the small sample size of this pilot cohort means that these preliminary conclusions need to be further validated, it clearly demonstrated a fit‐for‐purpose analytical workflow for studying FIELD samples.

### 4.6 Evaluation of quality control measures over time

We utilised book‐ended batching with an external plasma standard to monitor batch quality. We used the median area difference for the top 100 proteins, aiming to achieve <15% intra‐batch difference. On the occasions when this metric was exceeded, it prompted us to examine causes which were primarily due to declining MS detection sensitivity, or occasionally because of human errors in sample preparation pipetting. The sample preparation workflow itself is robust as we never experienced a failure using that procedure. For the LC‐MS using our setup with capillary flow it was typical to achieve 150 injections of 2 μg raw plasma digest before the system sensitivity deteriorated and failed batch QC. This was rectified by replacing the self‐packed 150 μm LC column, changing the transfer capillary and cleaning the MS front‐end ion optics to restore sensitivity, a process which would consume 1–3 days. This was a necessary process to enable quantitation of less abundant proteins, which would otherwise result in missing data.

In summary, we have reported a facile and robust human plasma sample preparation and LC‐MS workflow suitable for large‐scale proteomic analysis using neat plasma. The use of neat plasma has the advantage of avoiding complications due to additional sample processing steps, as well as time and reagent cost savings. We envisage this workflow which balances proteomic depth against cost and time can be applied to most clinical proteomic studies using human plasma where quantitative analysis of the major plasma proteins is the objective.

### 4.7 Associated data

Mass spectrometry data is available via ProteomeXchange with the identifier PDX029732.

## AUTHOR CONTRIBUTIONS

Matthew B. O'Rourke: Manuscript draft, carried out sample preparation, performed mass spectrometry and data analysis; Andrzej S. Januszewski: Critically reviewed data and manuscript draft; David R. Sullivan: Conceptualisation, funding acquisition, sample provision and study oversight; Imre Lengyel: Study design, validation; Alan J. Stewart: Methodology, validation; Swati Arya: Investigation, methodology; Ronald Ma: Funding acquisition; Sanjeev Galande: Funding acquisition; Anandwardhan A. Hardikar: Funding acquisition, supervision; Mugdha V. Joglekar: Investigation; Anthony C. Keech: Conceptualisation, funding acquisition, sample provision and study oversight; Alicia J. Jenkins: Conceptualisation, funding acquisition, sample provision and manuscript revision; Mark P. Molloy: Conceptualisation, methodology, funding acquisition, critical review of data and manuscript revision.

## CONFLICT OF INTEREST STATEMENT

The authors declare no competing interests.

## Supporting information

Supplementary Information

Supplementary Information

Supplementary Information

Supplementary Information

Supplementary Information

Supplementary Information

Supplementary Information

## Data Availability

Mass spectrometry data has been deposited at EBI‐PRIDE with the identifier PDX029732.
